# Effects of Astaxanthin on Reverse Cholesterol Transport and Atherosclerosis in Mice

**DOI:** 10.1155/2017/4625932

**Published:** 2017-11-01

**Authors:** Tang-Bin Zou, Shan-Shan Zhu, Fei Luo, Wei-Qiao Li, Xue-Rong Sun, Hong-Fu Wu

**Affiliations:** ^1^Dongguan Key Laboratory of Environmental Medicine, School of Public Health, Guangdong Medical University, Dongguan 523808, China; ^2^Guangdong Provincial Key Laboratory of Medical Molecular Diagnostics, Guangdong Medical University, Dongguan 523808, China; ^3^School of Basic Medical Sciences, Guangdong Medical University, Dongguan 523808, China

## Abstract

High plasma level of HDL-cholesterol (HDL-C) has been consistently associated with a decreased risk of atherosclerosis (AS); thus, HDL-C is considered to be an antiatherogenic lipoprotein. The development of novel therapies to enhance the atheroprotective properties of HDL may have the possibility of further reducing the residual AS risk. Reverse cholesterol transport (RCT) is believed to be a primary atheroprotective activity of HDL, which has been shown to promote the efflux of excess cholesterol from macrophage-derived foam cells via ATP-binding cassette transporter A1 (ABCA1), ATP-binding cassette transporter G1 (ABCG1), and scavenger receptor class B type I (SR-BI) and then transport it back to the liver for excretion into bile and eventually into the feces. In the current study, we investigated the effects of astaxanthin on RCT and AS progression in mice. The results showed that short- and long-term supplementation of astaxanthin promote RCT in C57BL/6J and ApoE^−/−^ mice, respectively. Moreover, astaxanthin can relieve the plaque area of the aortic sinus and aortic cholesterol in mice. These findings suggest that astaxanthin is beneficial for boosting RCT and preventing the development of AS.

## 1. Introduction

Epidemiological studies have shown that plasma HDL-cholesterol (HDL-C) concentration is inversely related to incidence of atherosclerosis [1–3]. Interventional studies in animals and humans have further demonstrated that raising plasma HDL-C level is effective in reducing the risk or severity of atherosclerosis (AS) [4, 5]. Further, HDL performs several antiatherosclerotic properties, such as antioxidation, anti-inflammation, antithrombus, and improves endothelial function [6]. However, the promotion of reverse cholesterol transport (RCT) is considered one of the most important antiatherosclerotic mechanisms by which HDL protects against AS [7]. The pool of RCT is composed of all tissues and cells of the body, including the arterial wall. Macrophages are the main cholesterol-loaded cell type in atherosclerotic lesions. Although macrophage RCT is a very small part of RCT, the efficiency of cholesterol efflux from cholesterol-loaded macrophages and macrophage-derived foam cells to feces plays a key role in protecting against AS progression [8]. Thus, the increase of macrophage RCT may serve as a potentially attractive approach for AS therapy [9]. Additionally, AS is an inflammatory disease that involves vascular and immune cell types. Endothelial cells, smooth muscle cells, and circulating leukocytes are the active players in atherosclerotic inflammatory processes [10]. Endothelial dysfunction is among the earliest events in the formation of AS. After stimulation with various inflammatory factors, endothelial cells upregulate intercellular adhesive molecule-1 (ICAM-1) and vascular cell adhesion molecule-1 (VCAM-1) expression via the activation of nuclear factor kappa B (NF-*κ*B). ICAM-1 and VCAM-1 are critical to the adhesion of circulating monocytes to the initial endothelial cell monolayer, which is followed by the formation of foam cells and AS in the arterial wall. Thus, the inhibition of ICAM-1 and VCAM-1 expression may be considered a good strategy to inhibit monocyte adhesion to endothelial cells and restrict early formation of AS [11].

Astaxanthin is a novel carotenoid nutraceutical occurring in many crustaceans and red yeasts [12]. It has exhibited various biological activities, including prevention or amelioration of cardiovascular disease, gastric ulcer, and diabetic nephropathy [13–15]. In this study, we established a model to determine the rate of macrophage RCT and investigated the effects of astaxanthin on RCT and the progression of AS in mice.

## 2. Materials and Methods

### 2.1. Materials

The RAW264.7 macrophages were obtained from the American Type Culture Collection (Manassas, VA). The ^3^H-cholesterol was obtained from Perkin Elmer (Waltham, MA). Oxidized LDL (ox-LDL) was prepared as described in a previous study [16]. Dulbecco's modified Eagle's medium (DMEM), penicillin-streptomycin, and fetal bovine serum (FBS) were obtained from Gibco BRL (Grand Island, NY). The C57BL/6J mice were purchased from the Animal Center of Sun Yat-sen University (Guangzhou, China). The ApoE^−/−^ mice were purchased from the Jackson Laboratory (Bar Harbor, ME). Total cholesterol (TC), triglycerides (TG), and HDL-C kits were purchased from BioSino Biotechnology Company (Beijing, China). Astaxanthin (purity ≥ 97%, from* Haematococcus pluvialis*) and dimethyl sulfoxide were obtained from Sigma-Aldrich (St. Louis, MO).

### 2.2. Establishment of RCT Model In Vivo

The RAW264.7 macrophages were cultured in DMEM supplemented with 10% FBS. Cells were radio-labeled with 5 *μ*Ci/mL ^3^H-cholesterol and cholesterol-loaded with 25 *μ*g/mL ox-LDL for 48 h. Then, 0.5 mL ^3^H-cholesterol-labeled macrophage-derived foam cell suspension (2.0 × 10^6^ cells) and 0.5 mL medium were intraperitoneally injected into C57BL/6J mice in the model and control group (*n* = 6), respectively. Finally, ^3^H-cholesterol levels in plasma, liver, and feces were measured by liquid scintillation counter [17, 18].

### 2.3. Effects of Astaxanthin on RCT in C57BL/6J Mice

Ten-week-old male C57BL/6J mice were divided into control and astaxanthin group (*n* = 6). Mice in the control and astaxanthin group were fed AIN-93G diet and AIN-93G diet plus astaxanthin (0.05%, w/w) for 2 weeks, respectively. At day 12, the mice were intraperitoneally injected with ^3^H-cholesterol-labeled macrophage-derived foam cells, and the rate of macrophage RCT was determined [19].

### 2.4. Effects of Astaxanthin on RCT and AS in ApoE^−/−^ Mice

Six-week-old male ApoE^−/−^ mice were divided into control and astaxanthin group (*n* = 6). Mice in the control and astaxanthin group were fed AIN-93G diet and AIN-93G diet plus astaxanthin (0.05%, w/w) for 12 weeks, respectively. Two days prior to the end of diet treatment, the rate of macrophage RCT was determined [19]. The plasma levels of TC, TG, and HDL-C were gauged by commercial kits. The atherosclerotic plaque area of the aortic sinus was evaluated after staining with Oil red O. The area of atherosclerotic plaque and lumen were calculated by automated image analysis software. The average ratio of atherosclerotic plaque to lumen was used to reflect the size of atherosclerotic plaque [20]. The cholesterol content of the thoracic and abdominal aorta was measured by enzymatic method [21].

### 2.5. Statistical Analysis

Results are presented as mean ± SEM. The data were statistically analyzed with the Student *t*-test. Probability values less than 0.05 were considered significant.

## 3. Results

### 3.1. Establishment of RCT Model In Vivo

A certain amount of ^3^H-cholesterol was detected in plasma of C57BL/6J mice throughout the experiment. Seen in Table 1, radioactivity was detected 6 h after intraperitoneal injection and reached a peak at 24 h and then declined until 48 h but remained higher than at 6 h. These results demonstrate that ^3^H-cholesterol could drain to plasma from macrophage foam cells and was effectively absorbed in the liver, leading to a decline of ^3^H-cholesterol in plasma. Seen in Table 2, ^3^H-cholesterol was found in the liver and feces after 48 h, which shows that ^3^H-cholesterol derived from macrophage foam cells was absorbed by the liver and was eliminated from the body along with feces, then completing the process of RCT. Hence, ^3^H-cholesterol levels in plasma, liver, and feces in the model group were significantly higher than the control group (*P* < 0.01).

### 3.2. Astaxanthin Increased RCT in C57BL/6J Mice

**Figure 1:**
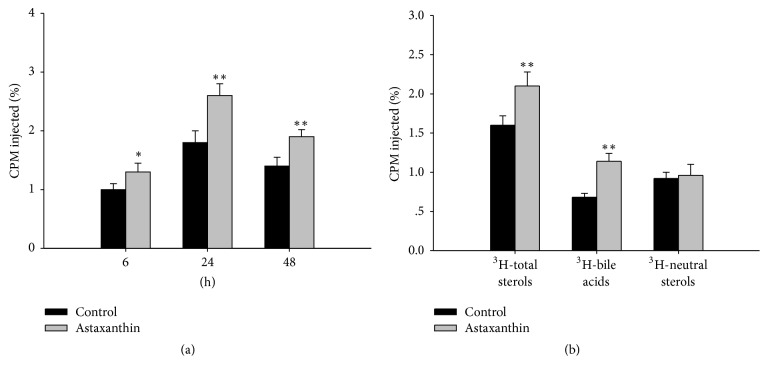
Effects of astaxanthin on RCT in C57BL/6J mice. (a) ^3^H-cholesterol level of plasma; (b) ^3^H-cholesterol level of feces (*n* = 6, ^*∗*^*P* < 0.05 versus control, ^*∗∗*^*P* < 0.01 versus control).

After the intervention of 12 days, ^3^H-cholesterol-labeled macrophage foam cells were intraperitoneally injected into C57BL/6J mice. The distribution of ^3^H-cholesterol in plasma is shown in Figure 1(a). The level of ^3^H-cholesterol in the astaxanthin group was significantly higher than the control group (*P* < 0.05, *P* < 0.01) after 6 h, 24 h, and 48 h. In addition, ^3^H-cholesterol-labeled macrophage foam cells were intraperitoneally injected into C57BL/6J mice after 48 h. The distribution of ^3^H-total sterols, ^3^H-neutral sterols, and ^3^H-bile acids in feces is shown in Figure 1(b). The levels of ^3^H-total sterols and ^3^H-bile acids in the astaxanthin group were significantly higher than the control group (*P* < 0.01), but there was no significant difference in ^3^H-neutral sterols in either group (*P* > 0.05). Further, ^3^H-cholesterol level in the liver was not significantly changed by treatment with astaxanthin (*P* > 0.05).

### 3.3. Astaxanthin Promoted RCT in ApoE^−/−^ Mice

**Figure 2:**
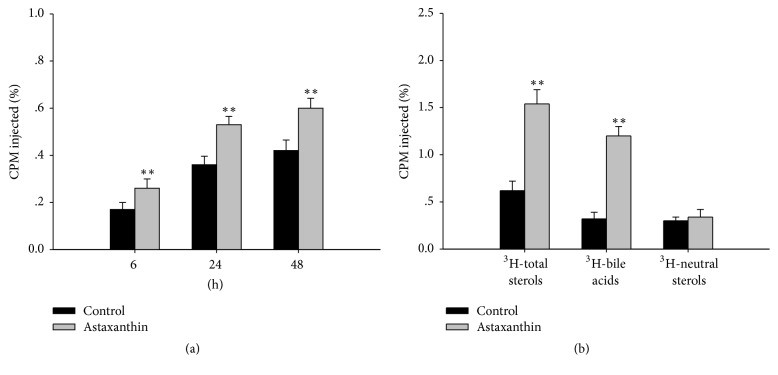
Effects of astaxanthin on RCT in ApoE^−/−^ mice. (a) ^3^H-cholesterol level of plasma; (b) ^3^H-cholesterol level of feces (*n* = 6, ^*∗∗*^*P* < 0.01 versus control).

After intervention of 12 weeks, ^3^H-cholesterol-labeled macrophage foam cells were intraperitoneally injected into ApoE^−/−^ mice. The distribution of ^3^H-cholesterol in plasma is shown in Figure 2(a). The level of ^3^H-cholesterol in the astaxanthin group was significantly higher than the control group (*P* < 0.01) after 6 h, 24 h, and 48 h. In addition, ^3^H-cholesterol-labeled macrophage foam cells were intraperitoneally injected into ApoE^−/−^ mice after 48 h. The distribution of ^3^H-total sterols, ^3^H-neutral sterols, and ^3^H-bile acids in feces is shown in Figure 2(b). ^3^H-total sterols and ^3^H-bile acids levels in the astaxanthin group were significantly higher than the control group (*P* < 0.01), but there was no significant difference in ^3^H-neutral sterols in either group (*P* > 0.05). Similarly, the level of ^3^H-cholesterol in the liver was not significantly changed by treatment with astaxanthin (*P* > 0.05).

### 3.4. Astaxanthin Inhibited Development of AS in ApoE^−/−^ Mice

After the intervention of 12 weeks, lipid levels in the ApoE^−/−^ mice were gauged. Seen in Table 3, the plasma levels of TC, TG, and non-HDL-C in the astaxanthin group were significantly lower than the control group (*P* < 0.05). However, the plasma level of HDL-C in the astaxanthin group was significantly higher than the control group (*P* < 0.05).

**Figure 3:**
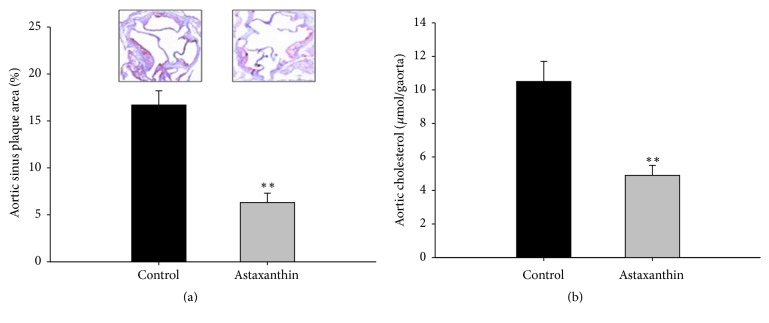
Effects of astaxanthin on AS in ApoE^−/−^ mice. (a) Relative plaque area of the aortic sinus; (b) cholesterol content of the aorta (*n* = 6, ^*∗∗*^*P* < 0.01 versus control).

Furthermore, the plaque area of the aortic sinus and the cholesterol content of the aorta in ApoE^−/−^ mice were measured. Seen in Figure 3(a), the relative plaque area of the aortic sinus in the astaxanthin group was significantly lower than the control group (*P* < 0.01), which indicates that astaxanthin effectively inhibited the development of AS. Seen in Figure 3(b), the cholesterol content of the aortas in the astaxanthin group was also significantly lower than the control group (*P* < 0.01).

## 4. Discussion

Accumulating evidence has shown that plasma HDL-C concentration is inversely correlated with incidence of atherosclerosis [22]. Clinical studies have further demonstrated that raising plasma HDL-C level is effective in reducing the risk of AS [23]. Further, HDL performs several antiatherosclerotic functions, such as antioxidaxion, anti-inflammation, and antithrombus, and improves endothelial function [6]. However, the promotion of RCT is thought to be one of the most important antiatherosclerotic mechanisms by which HDL protects against AS [7]. Our previous study reported that astaxanthin improved the plasma HDL-C level in C57BL/6J mice fed a high-fat diet [24]. Whether or not astaxanthin is beneficial to RCT in animal models remains unknown.

In the present study, we established a model to assess macrophage RCT in vivo as described in a previous study [19]. The levels of ^3^H-cholesterol in the liver and feces showed that ^3^H-cholesterol derived from macrophage foam cells can be absorbed by the liver and eliminated from the body along with feces, thus completing the process of RCT. Compared with the control group, ^3^H-cholesterol levels in plasma, liver, and feces were significantly higher in the model group, which shows that the macrophage RCT model was successfully established.

The effects of supplementation with astaxanthin on RCT in C57BL/6J mice were studied. After an intervention of 2 weeks, ^3^H-cholesterol level in plasma in the astaxanthin group was significantly higher than the control group after 6 h, 24 h, and 48 h. Meanwhile, ^3^H-total sterols and ^3^H-bile acids levels in feces in the astaxanthin group were significantly higher than the control group. However, there was no significant difference in ^3^H-neutral sterols in either group. From the above, it proves that astaxanthin can improve the efficiency of RCT in vivo. Interestingly, there was no significant difference in ^3^H-cholesterol level in the liver of the experimental group compared with the control group. Probably the ability of the liver to absorb ^3^H-cholesterol from plasma was reduced, or the ability of the liver to decompose ^3^H-cholesterol and secrete it into the bile, then out of the body along with feces, was enhanced.

In subsequent experiments, effects of supplementation with astaxanthin on RCT in ApoE^−/−^ mice were studied. After an intervention of 12 weeks, ^3^H-cholesterol level in plasma and ^3^H-total sterols and ^3^H-bile acids levels in feces were significantly enhanced by astaxanthin after 6 h, 24 h, and 48 h, but there was no significant difference in ^3^H-neutral sterols in either group. Likewise, there was no significant difference in ^3^H-cholesterol level in the liver of the experimental group compared with the control group. Obviously, the ^3^H-cholesterol level of plasma in ApoE^−/−^ mice is lower than C57BL/6J mice in the same time course. Further, astaxanthin obviously raised plasma HDL-C level and alleviated the plaque area of the aortic sinus and the cholesterol content of the aorta in the atherosclerotic ApoE^−/−^ mice. Hence, astaxanthin could inhibit the development of AS, which is consistent with a previous study [25].

Various enzymes and cholesterol transporters play crucial roles in the RCT pathway. Researchers have found that peripheral ATP-binding cassette transporter A1 (ABCA1) and ATP-binding cassette transporter G1 (ABCG1) are the main transporters for transferring cholesterol to plasma HDL, and hepatic scavenger receptor class B type I (SR-BI), plays a critical role in cholesterol uptake from plasma to the liver [26]. SR-BI mediates the exchange of cholesterol between HDL and cells and is a crucial factor in the transport of excessive cellular cholesterol from extrahepatic tissues to the liver and is also a factor in cholesterol homeostasis. Furthermore, plasma HDL-C is closely connected with ABCA1 and ABCG1 expression [27]. Iizuka et al. investigated the effects of astaxanthin on key molecules in cholesterol efflux from macrophages. They reported that astaxanthin did not modify peroxisome proliferator-activated receptor *γ* or liver X receptor levels, but it increased the expression of ABCA1 and ABCG1, thereby enhancing cholesterol efflux from macrophages [28]. However, the mechanism by which astaxanthin promotes RCT and inhibits the development of AS in vivo remains unknown and will be further researched in the future.

## 5. Conclusions

To summarize, the current study revealed that astaxanthin promotes RCT both in C57BL/6J and in ApoE^−/−^ mice. Moreover, astaxanthin can alleviate the plaque area of the aortic sinus and aortic cholesterol in mice. These findings suggest that astaxanthin is beneficial for boosting RCT and preventing the development of AS.

## Figures and Tables

**Table 1:** ^3^H-cholesterol distribution of C57BL/6J mice plasma in each group ($\overset{-}{x}\pm s$, *n* = 6).

^3^H cpm percent (%)	Control	Model
6 h	0.012 ± 0.001	0.973 ± 0.112^*∗∗*^
24 h	0.010 ± 0.002	1.690 ± 0.146^*∗∗*^
48 h	0.011 ± 0.002	1.445 ± 0.137^*∗∗*^

*Note*. ^*∗∗*^*P* < 0.01 versus control.

**Table 2:** ^3^H-cholesterol content of C57BL/6J mice liver and feces in each group ($\overset{-}{x}\pm s$, *n* = 6).

Group	^3^H cpm percent (%)			
	Liver	Feces total sterols	Neutral sterols	Bile acids
Control	0.008 ± 0.001	0.012 ± 0.002	0.007 ± 0.002	0.006 ± 0.001
Model	1.452 ± 0.128^*∗∗*^	1.578 ± 0.160^*∗∗*^	0.949 ± 0.097^*∗∗*^	0.635 ± 0.072^*∗∗*^

*Note*. ^*∗∗*^*P* < 0.01 versus control.

**Table 3:** Effects of astaxanthin on the lipid levels in the atherosclerotic ApoE^−/−^ mice ($\overset{-}{x}\pm s$, *n* = 6).

Group	TC (mmol/L)	TG (mmol/L)	HDL-C (mmol/L)	Non-HDL-C (mmol/L)
Control	13.95 ± 1.34	1.87 ± 0.12	0.64 ± 0.07	13.31 ± 1.48
Astaxanthin	10.46 ± 1.17^*∗*^	1.29 ± 0.15^*∗*^	0.92 ± 0.10^*∗*^	9.54 ± 1.36^*∗*^

*Note*. ^*∗*^*P* < 0.05 versus control.
